# Acupuncture and related interventions for anxiety in coronavirus disease 2019: A protocol for systematic review and meta-analysis

**DOI:** 10.1097/MD.0000000000021317

**Published:** 2020-07-24

**Authors:** Haowen Jia, Zhenzhen Han, Kai Zhang, Qilin Tang, Kaihang Sun, Hongwen Huang, Feng Qi

**Affiliations:** a Department of General surgery, Tianjin Medical University General Hospital, Tianjin, China; b Department of Acupuncture and Moxibustion, First Teaching Hospital of Tianjin University of Traditional Chinese Medicine, Tianjin, China School of Basic; c National Clinical Research Center for Chinese Medicine Acupuncture and Moxibustion; d Department of Acupuncture and Moxibustion, Tianjin Gong An Hospital, Tianjin; e School of Basic Medical Sciences, Hebei University of Chinese Medicine, Hebei, Shijiazhuang, China.

**Keywords:** acupuncture, anxiety, coronavirus disease 2019, protocol, randomized controlled trials, systematic review

## Abstract

**Background:**

Traditional Chinese medicine plays an irreplaceable role in the treatment and prevention of epidemic diseases in China. Acupuncture is an important part of Chinese medicine. During the coronavirus disease 2019 (COVID-19) epidemic, acupuncture and related interventions are used to treat COVID-19 patients in China. The systematic review aims to evaluate the efficacy and safety of acupuncture and relevant interventions for anxiety in COVID-19.

**Methods:**

We will search for randomized control and observational studies of acupuncture and related interventions for anxiety in COVID-19 in the 6 databases from inception to 31 October 2020. There is no language restriction. Two independent reviewers will screen and collect all trials, data extraction and evaluate the risk of bias of the researches. We will perform a meta-analysis if appropriate.

**Results:**

Our findings will evaluate the feasibility of acupuncture and related interventions as adjunctive therapy for anxiety in COVID-19 patients, which will be disseminated in a relevant conference and published in a peer-reviewed publication.

**Conclusion:**

Our research will appraise the overall quality and evidence of whether acupuncture and related interventions are effective therapies for anxiety in COVID-19.

## 1 Introduction

Recently, an outbreak of the highly infectious novel coronavirus disease 2019 (COVID-19) has swept globally.^[1]^ Scientists and doctors around the world are working on ways to treat or prevent COVID-19. Unfortunately, there is currently no specific antiviral therapy for this pandemic. The clinical manifestations of COVID-19 appear to be broad, including mild upper respiratory tract disease, severe pneumonia, and even death.^[1,2]^

In China, historical records indicate that traditional Chinese medicine (TCM) plays an indispensable role in the prevention and treatment of epidemics.^[3]^ Acupuncture is 1 of the TCM treatments. During the severe acute respiratory syndrome epidemic, the intervention of acupuncture has played a useful role.^[4]^ Clinical observation study found that acupuncture can relieve chest distress, fatigue, and other symptoms in patients with severe acute respiratory syndrome in convalescence.^[4]^ In the current Chinese medical system, TCM and western medicine are equally important, and TCM's participation in the treatment of COVID-19 patients is encouraged by the government.^[5]^ Chinese doctors and scientists have conducted many clinical studies on the treatment of COVID-19 with the combination of TCM and western medicine.^[3,6,7]^ It is worth noting that most COVID-19 patients in China have received integrated Chinese and western medicine. The comprehensive application of acupuncture, moxibustion, and Chinese herbal medicine has provided new ideas for the treatment and prevention of COVID-19.^[8]^ A retrospective observational study has shown that acupuncture (in conjunction with routine treatment) significantly improved chest pain, fatigue, insomnia, anxiety, nervousness, nausea, vomiting, and other symptoms in patients with COVID-19.^[9]^

Some of the intervention measures in clinical practice are a mixture of acupuncture and other relevant therapies, while acupuncture is delicately defined as stimulation of acupoints, with penetration into skin by thin metal needles. In other words, acupuncture and other relevant therapies also include moxibustion and transcutaneous electrical nerve stimulation.^[10]^ It is worth noting that the Chinese association of acupuncture and moxibustion has proposed and issued the guideline for acupuncture and moxibustion interventions on COVID-19 (Second edition).^[11]^ The main contents are shown in Table 1. Clinical observation of moxibustion on 42 cases of ordinary COVID-19 pneumonia showed that moxibustion can effectively relieve the negative emotions of COVID-19 patients, attenuate the symptoms of chest tightness and loss of appetite, with a high degree of acceptance.^[12]^ A randomized controlled study on intervention for the patients under quarantine after close contact with COVID-19 is undergoing. The study uses the internet and mobile phone software to remotely guide patients to moxibustion themselves, reducing the contact between doctors and patients.^[13]^

**Table 1:**
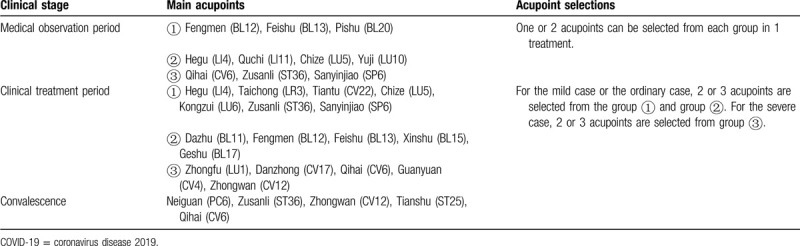
Main content of the guideline for acupuncture and moxibustion intervention on COVID-19.

**Figure 1:**
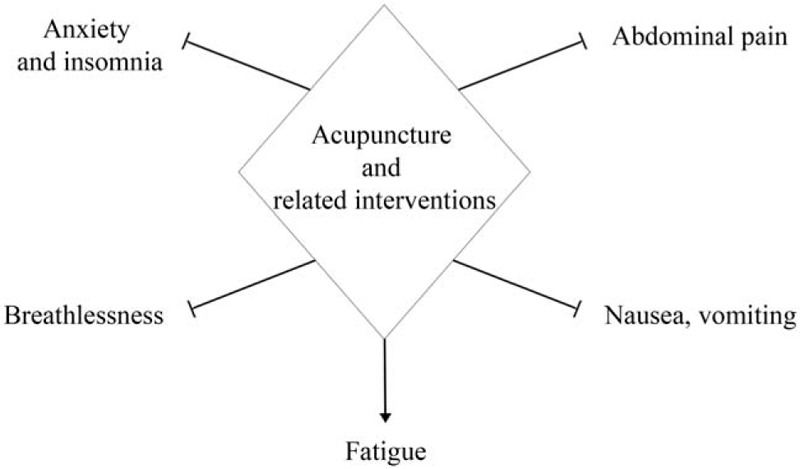
Possible related symptoms of COVID-19 treated with acupuncture. COVID-19 = coronavirus disease 2019.

**Figure 2:**
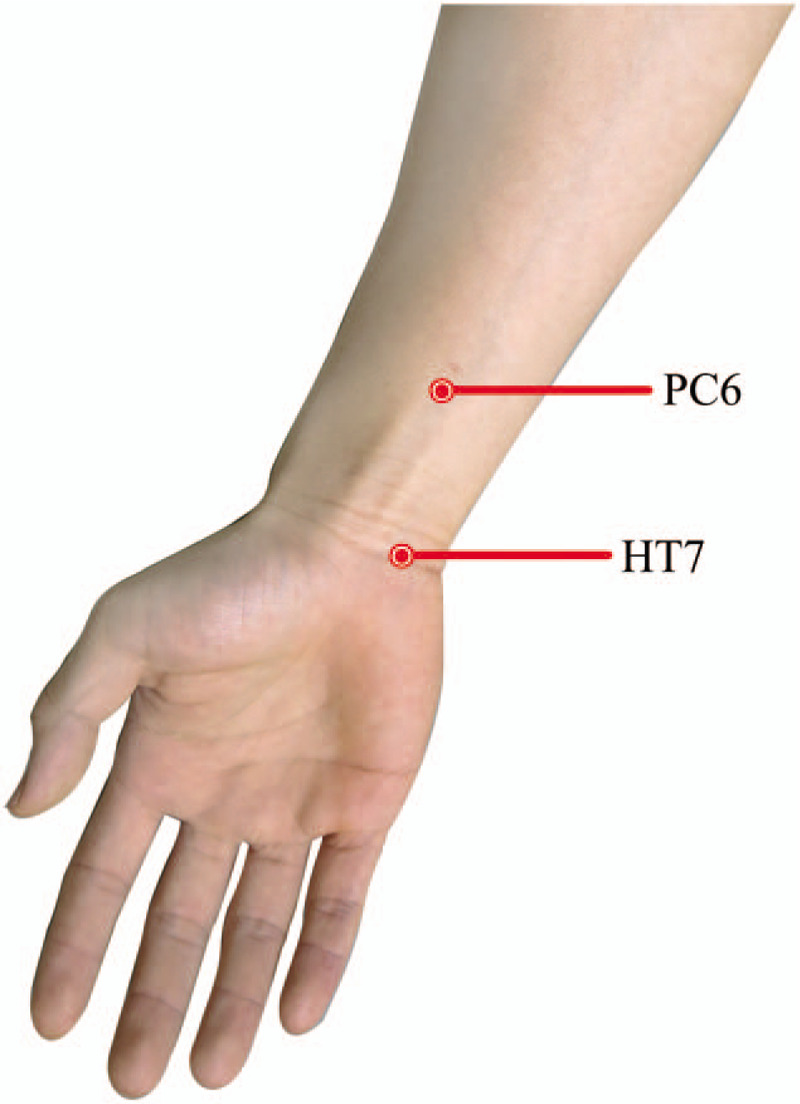
Acupoints commonly used in the treatment of anxiety in the arm.

The clinical presentation of COVID-19 has involved fever, cough, headache, abdominal pain, fatigue, and so on.^[2]^ During the COVID-19 epidemic, the general public (including COVID-19 patients) and healthcare workers are all likely to experience anxiety and insomnia.^[14,15]^ Acupuncture is an excellent symptom management therapy. Acupuncture and related interventions are often used to relieve common symptoms including vomiting, nausea, insomnia, dyspnea, fatigue, abdominal pain as well as anxiety disorders. ^[16–26]^ Possible related symptoms of COVID-19 treated with acupuncture is shown as Figure 1. A recent systematic review showed that acupressure has good results in relieving anxiety.^[27]^ Another systematic review revealed that there is good evidence to encourage the treatment of anxiety disorders with acupuncture, because it produces effective results and has fewer side effects than traditional therapies.^[28]^ Studies on data mining also summarizes the commonly used acupoints for anxiety treatment, as shown in Figure 2.^[29]^ Therefore, we intend to systematically review the clinical trials of acupuncture and related interventions for anxiety in COVID-19 patients. We aim to provide a more reliable evidence base for clinical practice in treating anxiety in COVID-19.

## 2 Methods

### 2.1 Study registration

Our research was registered in PROSPERO (CRD42020190153) on 5 June 2020. Also, this protocol report follows Preferred Reporting Items for Systematic Review and Meta-analysis Protocols guidelines.^[30]^

### 2.2 Inclusion criteria

#### 2.2.1 Types of studies

Randomized controlled trials (RCTs) and observational trials, including case control studies and cohort studies, that have tested acupuncture and related therapies interventions with or without western medicine for anxiety in COVID-19, will be included. Publications in any language will be included.

#### 2.2.2 Types of patients

Participants who were diagnosed as COVID-19 with anxiety will be included, without limits on age, gender, and race. The diagnosis of COVID-19 includes Chinese or international diagnostic criteria.^[31,32]^

#### 2.2.3 Types of interventions

Participants undergone acupuncture and related interventions treatment such as, electroacupuncture, acupuncture, fire needle, transcutaneous electrical nerve stimulation, auricular point therapy, and moxibustion will be included in the experimental group. There is no restriction on the duration.

Patients who have treated with interventions such as sham acupuncture, routine care, or conventional therapy will be included in the control group.

#### 2.2.4 Types of outcomes

The primary outcomes will be the therapeutic effects of treatment on anxiety. Both qualitative and quantitative outcomes, change in anxiety score from baseline to the last available follow-up, such as the state-trait anxiety inventory and shortened 10-item version of the state-trait anxiety inventory assessing the severity of anxiety will be allowed.^[33]^

The secondary outcomes will be evaluated based on adverse effects and adverse events.^[34–36]^ Furthermore, for COVID-19, which is an infectious disease, the risk of acupuncture application also needs to be considered. Therefore, the safety of physicians is also an outcome measure.

### 2.3 Search methods to identify studies

#### 2.3.1 Search strategy

We will search 6 electronic databases, including the VIP, China National Knowledge Infrastructure, Wan Fang Data, EMBASE, Medline as well as Cochrane Central Register of Controlled Trials (CENTRAL) from their inception till 31 October 2020. There will be no limitation on the language of the included studies. The combined use of medical subject headings and keywords is of considerable significance to the retrieval of related trials. Moreover, our searching strategy for Medline is shown in Table 2.

**Table 2:**
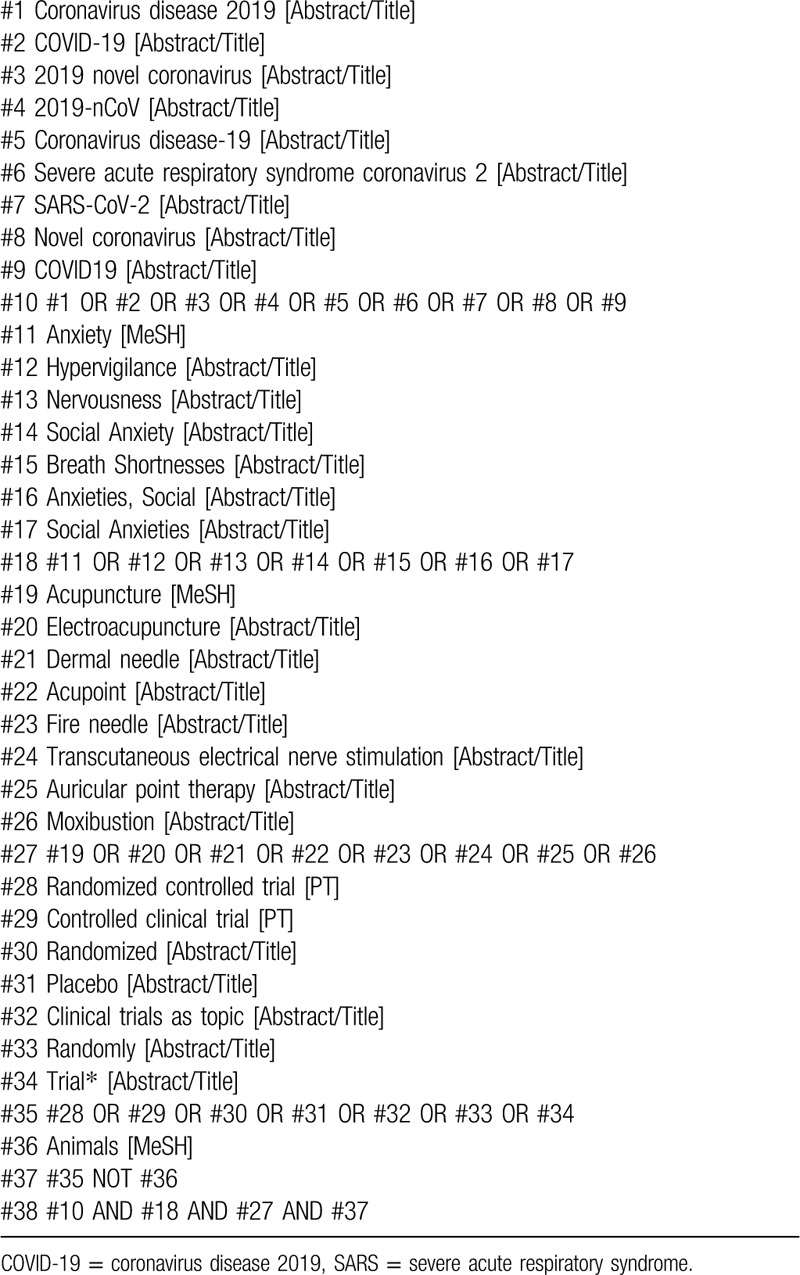
Search strategy for Medline.

### 2.4 Data collection and analysis

#### 2.4.1 Selection of studies

**Figure 3:**
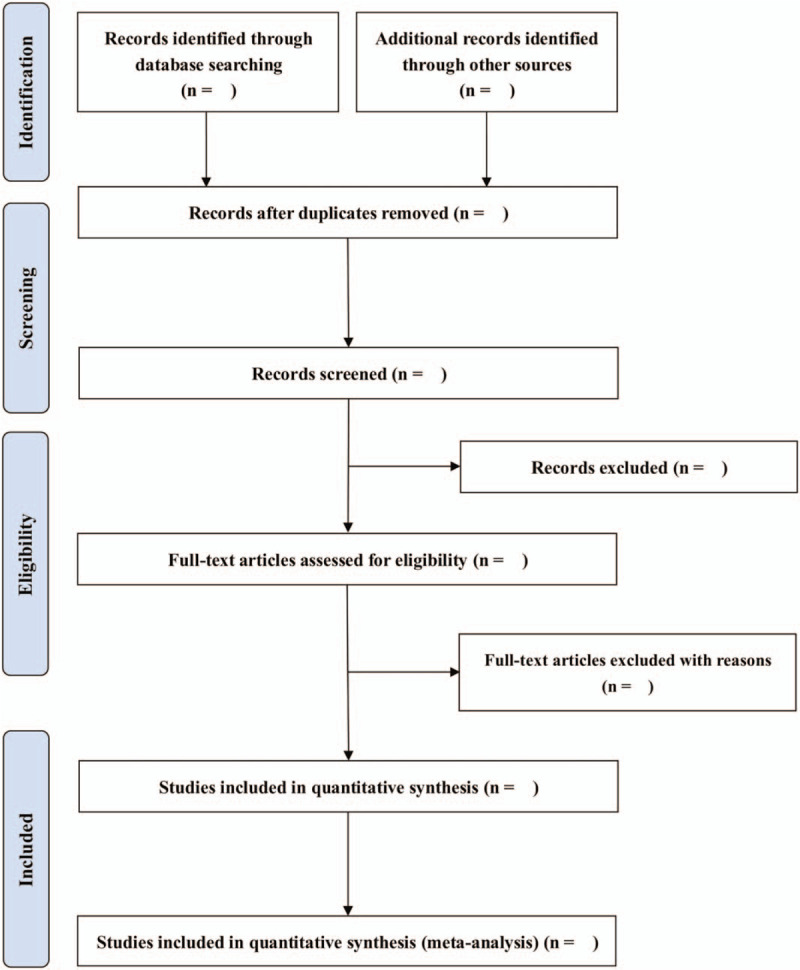
Study selection flow diagram.

Two independent researchers will generate and apply a standardized selection form to select appropriate researches. In the event of a disagreement, the third investigator will make the final decision. We will describe and summarize the selection process for each study in the Preferred Reporting Items for Systematic Review and Meta-analysis Protocols flowchart, with reasons for exclusion. The process of screening operation is shown in Figure 3.

#### 2.4.2 Data extraction and management

All data extraction will be independently undertaken by 2 investigators using predesigned forms, including study design, general information, characteristics of patients, comparison interventions, and outcomes. For studies with uncertain data, we will contact the authors by telephone or email to obtain the complete data. And studies without sufficient information will be excluded. If there is any dispute during data extraction, it will be referred to a third reviewer.

#### 2.4.3 Assessment of risk of bias

Two independent investigators will assess the risk of bias of included RCTs studies using the Cochrane Collaboration's risk of bias tool.^[37]^ And disagreements will be discussed with another reviewer. Each RCTs will be assigned as unclear risk, low, or high of bias for 7 domains (sequence generation, allocation concealment, blinding of personnel and participants, blinding of outcome assessors, incomplete result data, selective outcome reporting, and other biases), and a revised version of the Newcastle–Ottawa Scale for observational studies.^[38,39]^

#### 2.4.4 Measures of treatment effect

For continuous outcomes, data will be analyzed by using a weighted mean difference (WMD) or mean difference with 95% confidence intervals, while dichotomous data will be expressed as relative risk, with confidence intervals of 95%.

#### 2.4.5 Assessment of heterogeneity

Both the Chi-squared and *I*^2^ statistics will be used for the evaluation of heterogeneity, with an *I*^2^ of >50% or *P* < 0.1 considered indicative of high heterogeneity.

#### 2.4.6 Publication bias

If a sufficient number of trials are included, we will assess small study biases (such as publication bias) with funnel plots. For continuous variables, Egger tests will be recommended. For the dichotomous data, we will select the Harbord and Peters test.^[37]^

#### 2.4.7 Data synthesis

Stata, version 14.0 (StataCorp LLC), will be used to analyze the data. Studies that examine the same interventions and outcomes in similar populations will be combined using the meta-analysis to estimate the combined intervention effect. Heterogeneity among trials will be identified by the *I*^2^ and Chi-squared test statistics. If the included studies have no obvious heterogeneity (*I*^2^ < 50% or *P* > 0.1), we will use a fixed-effect model for calculation. Or else, a random-effects model will be used.^[37,40–43]^

#### 2.4.8 Sensitivity analysis

To test the robustness of the review findings, we will perform a sensitivity analysis for the primary outcome according to the statistical model.

#### 2.4.9 Subgroup analysis

If sufficient studies can be identified according to age, duration of treatment and different interventions, we will conduct a subgroup analysis.

#### 2.4.10 Assessment of evidence quality

We will use the Grading method of Recommendations Assessment, Development and Evaluation to rate the quality of evidence for each outcome as low, very low, moderate and high level.

### 2.5 Ethics

Ethical approval is not required because no individual patient or animal information is collected.

## 3 Discussion

Considering that COVID-19 is an important public health issue, the efficacy of acupuncture combined with routine treatment of COVID-19 remains inconclusive, and the findings of this research add some evidence to the knowledge in this field. The main challenge with this study is the generally poor quality of clinical studies on acupuncture.^[44–47]^ This research will provide evidence-based medicine to determine whether acupuncture and related interventions are effective interventions for patients with anxiety in COVID-19.

## Author contributions

**Conceptualization:** Feng Qi and Haowen Jia.

**Data curation:** Kai Zhang and Zhenzhen Han.

**Formal analysis:** Kai Zhang and Haowen Jia.

**Investigation:** Haowen Jia, Hongwen Huang and Kai Zhang.

**Methodology:** Kai Zhang and Hongwen Huang.

**Software:** Qilin Tang and Kaihang Sun.

**Supervision:** Qilin Tang.

**Writing – original draft:** Haowen Jia, Kai Zhang and Haowen Jia.

**Writing – review & editing:** Qilin Tang.
